# Explainable Artificial Intelligence for Human-Machine Interaction in Brain Tumor Localization

**DOI:** 10.3390/jpm11111213

**Published:** 2021-11-16

**Authors:** Morteza Esmaeili, Riyas Vettukattil, Hasan Banitalebi, Nina R. Krogh, Jonn Terje Geitung

**Affiliations:** 1 Department of Diagnostic Imaging, Akershus University Hospital, 1478 Lørenskog, Norway; hasan.banitalebi@ahus.no (H.B.); nina.rolland.krogh@ahus.no (N.R.K.); j.t.geitung@medisin.uio.no (J.T.G.); 2 Department of Electrical Engineering and Computer Science, Faculty of Science and Technology, University of Stavanger, 4021 Stavanger, Norway; 3 Faculty of Medicine, Institute of Clinical Medicine, University of Oslo, 0315 Oslo, Norway; dr.riyas@gmail.com; 4 Division of Paediatric and Adolescent Medicine, Oslo University Hospital, 0372 Oslo, Norway

**Keywords:** tumor localization, black box CNN, explainable AI, gliomas, machine learning

## Abstract

Primary malignancies in adult brains are globally fatal. Computer vision, especially recent developments in artificial intelligence (AI), have created opportunities to automatically characterize and diagnose tumor lesions in the brain. AI approaches have provided scores of unprecedented accuracy in different image analysis tasks, including differentiating tumor-containing brains from healthy brains. AI models, however, perform as a black box, concealing the rational interpretations that are an essential step towards translating AI imaging tools into clinical routine. An explainable AI approach aims to visualize the high-level features of trained models or integrate into the training process. This study aims to evaluate the performance of selected deep-learning algorithms on localizing tumor lesions and distinguishing the lesion from healthy regions in magnetic resonance imaging contrasts. Despite a significant correlation between classification and lesion localization accuracy (*R* = 0.46, *p* = 0.005), the known AI algorithms, examined in this study, classify some tumor brains based on other non-relevant features. The results suggest that explainable AI approaches can develop an intuition for model interpretability and may play an important role in the performance evaluation of deep learning models. Developing explainable AI approaches will be an essential tool to improve human–machine interactions and assist in the selection of optimal training methods.

## 1. Introduction

Artificial intelligence (AI) developments have created opportunities for human life in a wide range of industries, business, education, and healthcare [1,2]. As a part of AI, deep-learning-derived approaches provide convenient autonomous image classification in the medical domain [1]. Traditional modeling techniques such as linear regression and decision trees provide an understandable relationship between input data and the decisions in the model outputs [2]. These models are often called white-box models but are usually not as performant as black-box models such as convolutional neural networks (CNN), complicated ensembles, and other deep learning models. The latest models provide excellent accuracy at the expense of model explainability. Explainable models estimate the importance of each feature on the model predictions, providing interpretable tools for understanding deep learning outcomes [3]. Explainable AI is essential in tackling biases in AI-based decisions. Bias in models can originate long before the training and testing stages [3]. The data used for model training can be entangled in their own set of biases. Therefore, identifying and handling potential biases in datasets is a crucial component of any responsible AI strategy. AI-derived training should aim to build trustworthy, transparent, and bias-free models.

Magnetic resonance imaging (MRI) serves as a gold standard and is the method of choice for the in vivo investigation of most brain diseases, such as different types of brain tumors. In brain tumor examinations, several radiological characteristics, such as morphology, tumor volume and location, composition, and enhancement, can be derived to narrow the differential diagnosis and guide patient management. The most common MRI modalities in brain malignancy examinations include T1-weighted (T1w), T2-weighted (T2w), and Fluid Attenuation Inversion Recovery (FLAIR). These MR modalities provide distinctive imaging contrast to improve lesion identification and pathological delineations.

Deep learning methods have demonstrated unprecedented sensitivity and accuracy in tumor segmentation and malignancy classifications [4]. Several studies have explored explainable AI approaches to visualize the learning evolutions at each neuron layer [5,6]. The methods provide saliency maps to identify the contribution of pixels/features in the learning process. Explainable AI may improve the training performance of deep learning models by intermediate step monitoring at the inter-neuron layers [5,6]. Post hoc explainable evaluations of AI outcomes may profoundly contribute to the understanding of deep learning predictions and eventually enhance their transparency, reliability, and interpretability, and human–machine interactions [7,8]. Furthermore, post hoc methods may contribute to clinical neurophysiology and neuroimaging data analysis, such as the localization of brain sub-regions [9,10,11].

This study evaluates the high-level features of deep convolutional neural networks, predicting tumor lesion locations in the brain. We used explainable saliency maps to investigate the performance of three established densely deep learning models in accurately identifying tumor lesions.

## 2. Materials and Methods

The experiments include the TCGA dataset [12,13] retrieved from The Cancer Imaging Archive repositories [13,14,15,16]. The dataset consists of lower-grade gliomas and the most aggressive malignancy, glioblastoma (WHO grade IV). With the inclusion criteria of the availability of T2-weighted and FLAIR magnetic resonance (MR) images, we gathered MR data from 354 subjects. We analyzed the axial slices of the T2w images following preprocessing steps: (i) N4BiasCorrection to remove RF inhomogeneity, (ii) intensity normalization, (iii) affine registration to MNI space, and (iv) resizing all images to the resolution of 256 × 256 × 128 using ANTs scripts [16]. A total number of 19,200 and 14,800 slices of brain images with and without tumor lesions, respectively, were prepared for training.

We trained different AI networks with three-fold cross-validation by randomly shuffling imaging data for training, validation, and testing patches. Three identical groups with dataset distributions of 55%, 15%, and 30% for training, validation, and testing, respectively, were generated. Each fold of cross-validation and testing was a new training phase based on an identical combination of the three groups. We held out the testing fraction of the dataset from the in-training step. Thus, the accuracy of the models was calculated using the mean value of the network performance on only the testing dataset.

Selecting our choices from the most deployed AI models in the imaging domain, we included the AI networks DenseNet-121 [17], GoogLeNet [18] (also known as Inception V1, available in Github-GoogLeNet in Keras), MobileNet [19] (retrieved directly from Keras-https://keras.io/api/applications/mobilenet/ (accessed on 11 June 2020)). We incorporated the data from T2w and FLAIR sequences as separate channel inputs to each model, similar to the process of handling RGB inputs. Thus, each input slice contained combined MR images of T2w and FLAIR. Each model had a 2-class output consisting of healthy and tumor. All models employed Adam optimizer with a learning rate of 5*e*–4, with a decay rate of 0.9. The batch size and number of epochs were 25 and 100, respectively. All experiments were computed on a computer with two Intel Corei7 CPU, Nvidia GeForce Quadro RTX GPU, and 32 GB of memory. We implemented the Grad-CAM algorithm to visualize each model’s performance on tumor lesion localization [6]. The Grad-CAM generates visual explanations of post-processed image space gradients using saliency heatmaps. The normalized heatmap (SanityMap ϵ [0 1]) scales the contribution of each pixel in the learning process, ranging from none (“0”) to the most significant (“1”) pixels in the 2-dimensional image. This study determined the most important pixels with a cuff-off value greater than “0.5” in the SanityMap. The overlap of the generated heatmaps ([SanityMap] ≥ 0.5) and the tumor lesions in one testing group was used to estimate the localization performance as Equation (1):

(1) $$Localization_{hit}\left(\%\right)=\frac{hits}{total\;testing\;images}\;$$

where *hits* was determined as a successful overlap of >50% of pixels of the tumor mask with the Grad-CAM-derived heatmap ($SanityMap_{0.5}\cap Tumor_{mask}>50\%$). We also calculated the Intersection over Union (IoU) metric as Equation (2) (the Jaccard index) for localization accuracy. IoU is a popular metric for segmentation and object detection [20].

(2) $$IoU\left(\%\right)=\frac{SanityMap_{0.5}\cap \;Tumor_{mask}}{SanityMap_{0.5\;}\;\cup \;Tumor_{mask}}\;$$

Statistics: The mean difference between the computed localization accuracy of each model was compared using the Mann–Whitney test. Spearman correlation analysis was calculated to investigate the relationships between the model’s prediction accuracy and tumor localization. The threshold for statistical significance was defined as *p* < 0.05.

## 3. Results

**Figure 1:**
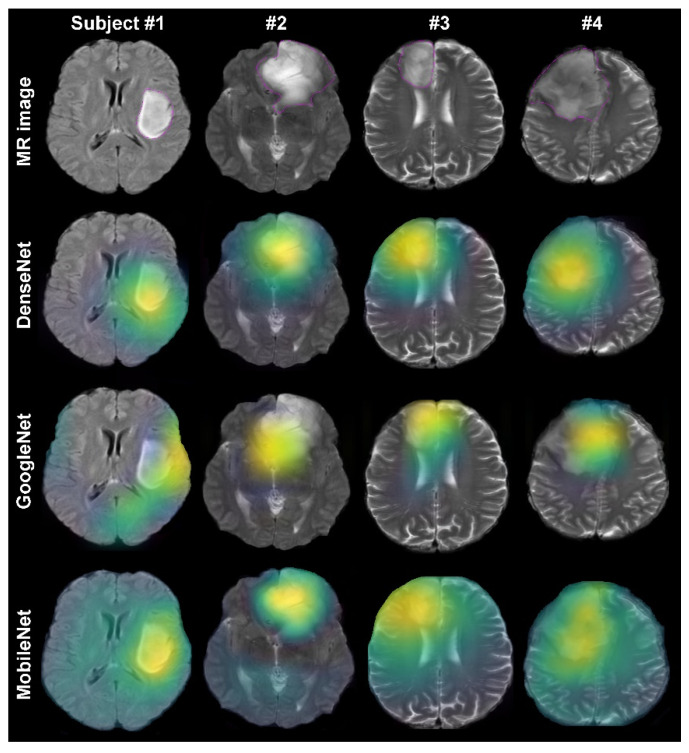
Heatmap visualization overlaid on tumor lesions for different training networks. The top row depicts the original MR image examples from four subjects. The magenta counters indicate the tumor lesion boundaries. The bottom rows show the Grad-CAM visualizations for three different training algorithms on the selected axial slices.

The Grad-CAM method provided representative heatmaps, which can be used to quantitively and qualitatively investigate the performance of convolutional neural networks. Examples of heatmaps from four subjects are shown in Figure 1. The DenseNet-121, GoogLeNet, MobileNet achieved a mean cross-validated prediction accuracy of 92.1, 87.3, and 88.9, respectively, on the testing dataset (Table 1). DenseNet-121 provided a significantly higher mean localization accuracy of *hit* = 81.1% and IoU = 79.1% than GoogLeNet (*p* = 0.01) and MobileNet (*p* = 0.02) (Table 1). The correlation analysis showed a significant agreement between the models’ prediction accuracy and specificity in localizing tumor lesions (*R* = 0.46, *p* = 0.005).

## 4. Discussion

Our study demonstrated that the explainable Grad-CAM method could visualize the network’s performance, distinguishing the images with and without tumor based on the lesion’s localization rather than other features in the brain. However, the results showed that a considerable number of tumor brains were classified by false-positive features, identifying other important components in the brain than the tumor lesion. The results indicated that, among the implemented three models, the DenseNet-121 performed better than the other two models, providing more accurate tumor localization (with ~80% hits and IoU). Similarly, previous studies in brain tumor segmentation and classification showed the state-of-the-art performance of the DenseNet [17,21,22,23], with an accuracy greater than 90%.

Grad-CAM provides post hoc feature attribution heatmaps, aiming to explain the relationship between the models’ predictions and in terms of its features. Operationally, the method employs gradients as a measure of importance in the feature space and may be used to explore significant features in the training phase, identifying data skew, or debugging model performance. Several explanatory CAM approaches have recently been proposed in the medical domain [24]. For example, Vinogradova and colleagues [25], produced visual explanations for semantic segmentation by extending Grad-CAM. The authors examined their method on the performance of a U-Net model in feature extraction of Cityscapes dataset and showed that the initial convolutional layers exhibit low-level edge-like feature extractions. Grad-CAM based approaches were also used to visualize 2-dimensional [6,26] and 3-dimensional brain tumor segmentation [27].

Already available in the Keras method libraries, Grad-CAM methods have demonstrated great capabilities in image region discrimination in various clinical and computer vision studies [25,26,27,28]. Despite the utility of Grad-CAM, there are some limitations associated with gradient-based explanation and Grad-CAM estimations, especially when targeting multiple objects in an image. Several studies have addressed the gradient-based visualization issues and suggested an extended version of CAM approaches, such as pyramid gradient-based class activation map [29] and high-resolution CAM, to target multiple organs in the computed tomography (CT) scan database [30]. However, Grad-CAM provided acceptable accuracy for our study because we aimed to target only one feature, i.e., tumor lesion, on 2-dimensional MR images.

T2-weighted and FLAIR sequences are MRI acquisition protocols that are recommended by neuro-radiologists for use in brain tumor clinical investigations [31]. The combination of T2w and FLAIR contrast effectively contributed to the performance of deep learning models, as cerebrospinal fluid can be distinguished from edema in these images [32]. The consensus recommendations for standardized brain tumor studies [31] for clinical trials have included functional imaging data acquisitions such as diffusion-weighted imaging. Diffusion imaging provides an apparent diffusion coefficient (ADC) metric, which can be included as an extra layer for deep learning analysis. Since ADC is a sensitive diagnostic metric providing cellular density maps [31], it would be interesting to investigate its contribution to future deep learning training protocols for classification or segmentation tasks.

Despite the close accuracy scores (greater than 90% accuracy) on the ImageNet database achieved by GoogLeNet, MobileNet, DenseNet, and several other complex models, explainable algorithms have shown faulty reasoning and feature extractions [5,6]. In other words, the machine interprets the image features differently, beyond human interpretation and recognition. In cancer clinics, the interpretation of radiological images plays a crucial role in clinical decisions. Thus, explainable AI methods can increase the translational potential of the developed complex models into clinics. The visual explanation of explainable AI provides deep learning algorithms’ reasoning and learning process in initiative ways that reflect the model’s final output. The visual interpretation will eventually help the investigators and clinicians to examine the true-positive and false-positive outputs achieved by CNN and debug the training pipeline. A platform with an interaction between the end-user and the CNN-based black-box can relate the trained model prototyping to what humans describe as logical components in classifying images. Such a platform generates a prototypical part network to interpret trained models in classification tasks [5].

Visual explanation techniques may potentially contribute to debugging model performance, selecting training strategy or architecture, and training workflow [3]. Wang and colleagues [33] incorporated explainable methods during training. The authors assess the utility of implementing features to increase explainability early in the development process. Chen and colleagues [5] showed that integrating interpretable methods can increase up to 3.5% in the accuracy score of trained models. Zhang and colleagues [34] integrated a Grad-CAM derived heatmap into their deep learning models to develop an effective strategy for identifying the brain areas underlying disease development in multiple sclerosis. A future work could be the inclusion of explainable AI approaches in the training phase to enhance both model classification and localization accuracy.

We performed this study on limited patient data, and thus trained the CNN models on slice-level. In future studies, with access to more patient data, we aim to examine our approach at the patient-level and perform a 3D analysis of tumor localization. Additionally, a prospective investigation may include clinical covariants, such as tumor volume, position, grade, and age, in the deep learning analysis. Indeed, a large range of patient data from multiple centers integrated with novel transfer learning approaches will be essential for such investigation.

## 5. Conclusions

In summary, we used a well-known explainable AI algorithm to evaluate the performance of three deep learning methods in localizing tumor tissues in the brain. Our results show that the incorporation of explainable AI in deep learning workflows plays an essential role in human–machine communication and may assist in the selection of an optimal training scheme for clinical questions and the AI learning progress.

## Figures and Tables

**Table 1:** Mean classification and localization accuracy on the testing database for DenseNet, GoogLeNet, and MobileNet.

Model	Classification	Localization	
	Accuracy (%)	Hits (%)	IoU (%)
**DenseNet-121**	92.1	81.1	79.1
**GoogLeNet**	87.3	73.7	73.8
**MobileNet**	88.9	77.8	76.7

## Data Availability

The TCGA data are available for research purposes (https://www.cancer.gov/tcga (accessed on 6 February 2020)). The deep learning codes are available on the TensorFlow database (accessed on 11 June 2020).
